# Combination Topical Antioxidant Protocol Enhances Laser‐Induced Improvement in Facial Pigmentation and Photoaging: A Prospective, Split‐Face Trial

**DOI:** 10.1111/jocd.70994

**Published:** 2026-06-19

**Authors:** Jihee Boo, Jamal Mohammed Alqahtani, Hyun Kim, Shinwon Hwang, Patricia Brieva, Jemin Kim, Jihee Kim

**Affiliations:** ^1^ Department of Dermatology, Yongin Severance Hospital Yonsei University College of Medicine Gyeonggi‐do South Korea; ^2^ Department of Dermatology, Severance Hospital, Cutaneous Biology Research Institute Yonsei University College of Medicine Seoul South Korea; ^3^ Department of Dermatology Imam Abdulrahman Bin Faisal University Dammam Saudi Arabia; ^4^ SkinCeuticals New York New York USA

**Keywords:** antioxidants, laser treatment, pigmentation, post‐procedural care

## Abstract

**Background:**

Laser‐based procedures are commonly used for facial pigmentation, and post‐procedural skincare is considered an important factor influencing outcomes. However, evidence remains limited regarding whether recovery‐focused skincare provides additional benefit when used with topical antioxidants after laser treatment.

**Aims:**

To compare the clinical efficacy of an integrated post‐procedural skincare regimen combining an antioxidant serum with a recovery cream versus antioxidant treatment alone following laser treatment for facial pigmentation and photoaging.

**Patients/Methods:**

This prospective, randomized, single‐blind, split‐face trial included 26 adults with clinically evident facial pigmentary lesions and signs of photoaging. Participants underwent two sessions of picosecond 1064‐nm Nd:YAG and CO_2_ laser treatment at two‐week intervals. Both sides received a topical antioxidant serum, while one randomized side additionally received a recovery cream for 8 weeks. Outcomes were evaluated using the modified Griffith's scale and hemi‐MASI. Biophysical parameters, including melanin index, erythema index, elasticity, and hydration, were measured using noninvasive instruments. Safety was assessed throughout the study.

**Results:**

At Week 8, the combination‐treated side showed greater improvement in modified Griffith's scale scores (29.3% vs. 19.4%, *p* = 0.04) and hemi‐MASI scores (37.2% vs. 20.3%, *p* = 0.02) compared with antioxidant treatment alone. Improvement was observed as early as Week 2 on the combination‐treated side. Both groups demonstrated significant reductions in melanin index with earlier improvement in the combination group. No serious adverse events were observed.

**Conclusions:**

The addition of recovery‐focused skincare to antioxidant post‐procedural care resulted in greater and earlier improvement in facial pigmentation following laser treatment compared with antioxidant alone.

## 1 Introduction

Lasers and light‐based devices are widely used to treat facial hyperpigmented lesions, including solar lentigines, melasma, and post‐inflammatory hyperpigmentation (PIH) [1, 2, 3]. However, pigmentary disorders remain challenging to manage because of their multifactorial pathogenesis, chronic course, and high recurrence rates, and iatrogenic dyspigmentation following laser procedures continues to be a major concern, particularly in patients with skin of color [4, 5].

PIH is a common sequela of cutaneous inflammation, trauma, and aesthetic procedures and represents one of the most frequent complications after laser treatment [4, 5]. Its reported incidence varies depending on laser modality, ranging from 6.7% to 53% after 532‐nm Q‐switched Nd:YAG laser treatment in Asian patients and reaching up to 92% following ablative fractional resurfacing in Fitzpatrick skin type IV [3]. PIH typically peaks approximately 4 weeks after treatment [3]. The development and persistence of PIH are driven by a complex interplay of epidermal injury, inflammatory mediators, oxidative stress, and melanocyte activation. Accordingly, peri‐procedural strategies aimed at reducing inflammation and restoring the epidermal barrier are emphasized in clinical practice; however, standardized, evidence‐based post‐procedural protocols remain limited [4, 5, 6].

The use of adjuvant topical cosmeceuticals has increased to improve laser outcomes and reduce procedure‐related complications. Vitamin C (L‐ascorbic acid) is a representative example, acting as a potent antioxidant that scavenges reactive oxygen species, inhibits tyrosinase activity, and promotes collagen synthesis, thereby supporting dermal remodeling and reducing melanogenesis [7, 8]. A split‐face study reported that Asian patients who applied a topical antioxidant serum containing vitamin C, vitamin E, and ferulic acid immediately after laser treatment showed faster wound recovery and reduced post‐laser erythema [7]. In addition, application of the same antioxidant serum following Q‐switched 1064‐nm Nd:YAG laser treatment for environmentally induced pigmentation was shown to be safe, well tolerated, and clinically effective, supporting its role as an adjunctive treatment for laser‐based pigmentation and photoaging management [9]. Furthermore, delivery of vitamin C via sonophoresis in combination with Q‐switched Nd:YAG laser treatment in Asian patients with dyschromia and melasma resulted in marked improvement in pigmentation, with reported reductions exceeding 75%, along with a substantial decrease in PIH [10].

Despite these encouraging findings, post‐laser pigmentation outcomes are influenced not only by antioxidant defense but also by the speed of epidermal barrier recovery and the degree of procedure‐induced inflammation, particularly in individuals at higher risk for PIH. Accordingly, topical strategies that address both barrier restoration and inflammatory modulation may offer additional benefit beyond antioxidant treatment alone.

An integrated approach incorporating antioxidant skincare, barrier‐repair support, careful pretreatment risk assessment, optimized laser parameters, and standardized peri‐procedural care has therefore been proposed to improve clinical outcomes while minimizing pigmentary complications. In this context, the present study was designed to evaluate the efficacy of an integrated antioxidant skincare regimen as an adjunct to picosecond 1064‐nm Nd:YAG and CO_2_ laser treatments for pigmentary lesions.

## 2 Materials and Methods

### 2.1 Study Design and Participant Selection

We conducted a prospective, randomized, single‐blind, split‐face trial to evaluate the effectiveness of combination antioxidant formulations (Regen6 and CE Ferulic, SkinCeuticals Inc.) as adjunctive therapy to laser treatment for improving facial pigmentation and photoaging. The study enrolled 26 adults aged 20–65 years with Fitzpatrick skin types III (*n* = 22) and IV (*n* = 4), presenting with clinically evident facial aging signs, including pigmentary lesions (melasma, solar lentigines, and ephelides), rhytides, and decreased skin elasticity. Participants with a documented history of keloid formation, pregnancy, psychiatric disorders, or any facial treatments (such as chemical peels, surgical procedures, or energy‐based devices) within the past 6 months were excluded. Additionally, none of the participants were receiving hormonal therapy or other medications that influence skin pigmentation during the study period.

Randomization of treatment assignments was performed using computer‐generated sequences (Microsoft Excel 2019; Microsoft, Redmond, WA, USA). Each side of the face was systematically randomized to receive either the single or the combination antioxidant formulations following laser procedures. The study protocol was approved by the Institutional Review Board of our institution (IRB No. 9–2025‐0014), and all participants provided written informed consent before enrollment.

### 2.2 Test Product and Treatment Regimen

Treatment was performed using a picosecond 1064‐nm Nd:YAG laser (PicoWay, Candela, USA) and a CO_2_ laser system (SNJ‐1000 U, SNJ Medical, Korea). All procedures were performed by a single board‐certified dermatologist (JHK). Before each treatment session, a topical anesthesia consisting of a 2.5% lidocaine hydrochloride and 2.5% prilocaine cream mixture was applied under occlusion for 30 min. The Nd:YAG laser was applied to the pigmented lesions in overlapping passes (532 nm, 0.3–0.4 J, 7 Hz; 1064 nm 0.8–1.2 J, 7–10 Hz), immediately followed by the CO_2_ laser for the focal hyperkeratotic lesions. Laser parameters were individually adjusted based on each patient's skin condition and the specific treatment area. Laser parameters for the major clinical indications have been described in greater detail in the Methods section, and a summary of indication‐specific settings has been added as a Supplementary Table S1.

Participants underwent two laser treatment sessions separated by two‐week intervals. Starting 2 weeks before the initial laser session, each participant followed a standardized daily skincare protocol. In the morning routine, participants applied 4–5 drops of CE Ferulic serum (containing vitamin C, vitamin E, and ferulic acid; SkinCeuticals Inc.) to the entire face until fully absorbed, followed by the application of one pump of either Regen6 antioxidant cream (containing niacinamide, ectoin, ceramide analogues, signal peptides, and botanical‐derived ingredients; SkinCeuticals Inc.) or a basic moisturizer without antioxidant components to the randomly allocated side of the face. Subsequently, the provided sunscreen was applied to the entire face. In the evening routine, participants applied one pump of either Regen6 antioxidant cream or basic moisturizer to their assigned facial side. After each laser treatment, participants continued this daily application protocol for a total of 8 weeks.

To reduce cross‐contamination, subjects were instructed to carefully apply products to each side without crossing the midline. Furthermore, oral antioxidant and nutritional supplements were banned throughout the study to prevent potential systemic confounding effects. Also, all subjects were instructed to maintain this regimen consistently throughout the entire study period. Compliance with the treatment protocol was verified through patient‐maintained diaries and regular monitoring of product usage volumes. The study lasted 8 weeks, including 4 clinical assessment visits: baseline (Week 0, pre‐laser evaluation), Week 2 (first laser treatment), Week 4 (second laser treatment), and Week 8 (final assessment). For analysis and presentation, the treatment groups were designated as the CEF + RGN6 group (receiving CE Ferulic serum followed by Regen6 antioxidant cream) and the CEF group (receiving CE Ferulic serum followed by a basic moisturizer without antioxidant components).

### 2.3 Clinical Assessment

At each scheduled visit, two board‐certified dermatologists (JMK and SWH), who remained blinded to treatment allocation, independently evaluated the clinical efficacy parameters. Assessments were done using standardized digital photographs and a three‐dimensional skin imaging system (Antera 3D, Miravex, Dublin, Ireland), with images presented in randomized, non‐chronological order.

Treatment outcomes were measured with two main assessment scales. First, the clinical evaluation of overall photodamage—including coarse and fine wrinkles, skin tone evenness, and tactile roughness—was performed using the modified Griffith's scale [11]. Second, hemi‐MASI (Melasma Area and Severity Index) scores were calculated to assess melasma severity on each half of the face [9]. This dual‐assessment approach, using both the modified Griffith's scale and the hemi‐MASI, has been similarly employed and validated in a recent split‐face trial of comparable design conducted by the same investigators [12]. Each investigator evaluated the parameters independently, and average values were used for statistical analysis. Additionally, a patient satisfaction questionnaire was administered at each visit using a 5‐point scale (Grade 1 = worsening; Grade 2, 0%–25% = minimal improvement or no change; Grade 3, 26%–50% = moderate improvement; Grade 4, 51%–75% = marked improvement; Grade 5% > 75% = near‐complete improvement).

### 2.4 Skin Biophysical Parameters

Objective skin biophysical parameters were measured using non‐invasive instrumentation to complement physician assessments. The Mexameter MX18 (Courage Khazaka, Köln, Germany) quantified skin erythema and pigmentation through erythema and melanin indices. Skin elasticity was evaluated with the Elastometer EM 25 (Courage Khazaka), while skin hydration was measured using the Corneometer CM 825 (Courage Khazaka). All measurements were taken at baseline (Week 0) and during follow‐up visits at Weeks 2, 4, and 8. All biophysical measurements were performed under standardized environmental conditions, maintained at a temperature of 20°C–24°C with relative humidity of 45%–55%. Participants acclimated to the climate‐controlled room for at least 20 min before each evaluation. To prevent confounding factors, all subjects were instructed to avoid applying any topical products, including the study serum, placebo, and moisturizers, for at least 8 h before each assessment. Measurements were performed in triplicate from consistent, predetermined locations on both cheeks at each time point, with average values used for statistical analysis.

### 2.5 Safety and Tolerability Assessment

Safety evaluations were conducted at each study visit through comprehensive visual inspections and physical exams. Treatment‐related adverse events, such as erythema, edema, hyper‐ or hypopigmentation, punctate hemorrhage, ecchymosis, and scarring, were systematically recorded based on both participant reports and investigator observations.

### 2.6 Statistical Analysis

Data are presented as frequencies (percentages) or means ± standard deviations, depending on the variable type. The primary statistical analysis used was a repeated‐measures analysis of variance (RM‐ANOVA) to evaluate changes over time for each parameter and to compare differences between treatment sides. For time‐dependent comparisons between groups, post hoc analyses with Student's *t*‐tests and Bonferroni correction were performed to examine parameters at individual timepoints. Statistical significance was set at *p* < 0.05. All statistical analyses were conducted using Python version 3.9.7 and R version 4.1.3.

## 3 Results

### 3.1 Demographics and Subject Disposition

A total of 26 participants (21 females and 5 males; mean age 46.9 ± 10.6 years; age range 33–65 years) were enrolled from April 2025 to August 2025. At baseline evaluation, the modified Griffith's scale yielded a mean score of 23.2 ± 5.14 points, with individual scores ranging from 15 to 35 points.

All 26 participants completed the entire 8‐week study protocol without any dropouts or missing data at any time point. Analyses of skin aging parameters, safety outcomes, and participant satisfaction questionnaires were performed using data from all enrolled participants across all assessment visits (baseline, Weeks 2, 4, and 8).

### 3.2 Clinical Assessment of Skin Aging Parameters

**FIGURE 1:**
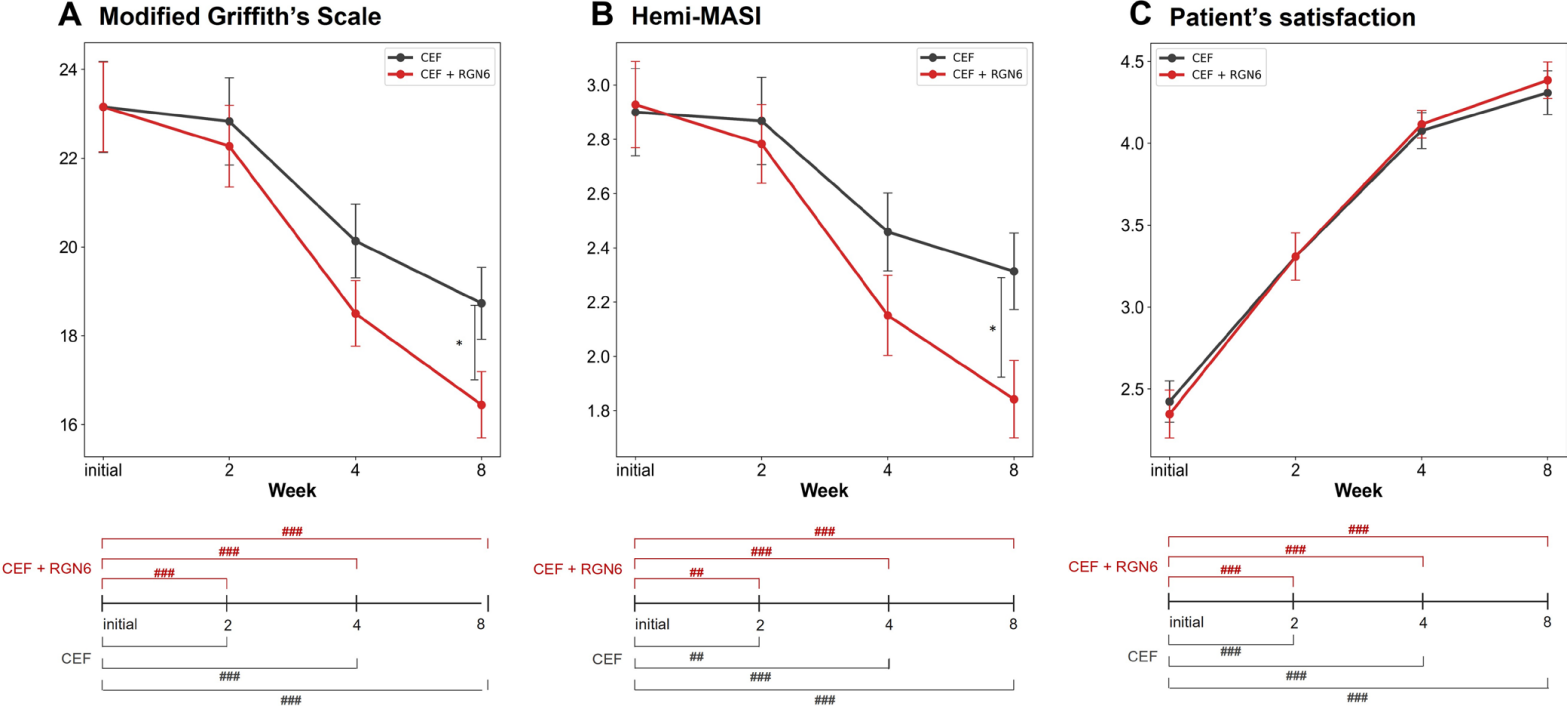
Temporal changes in clinical parameters during 8 weeks of treatment: (A) Modified Griffith's Scale Scores, (B) Hemi‐MASI (Melasma Area and Severity Index) Scores, and (C) Patient satisfaction questionnaire. Values represent mean ± standard error. Upper graphs illustrate changes in clinical parameters over time, whereas lower bars indicate the statistical significance of differences between baseline and each time point. *p < 0.05, **p < 0.01, ***p < 0.001 for comparisons between CEF and CEF + RGN6 groups at each time point with Bonferroni correction, and #*p* < 0.05, ##*p* < 0.01, ###*p* < 0.001 for within‐group comparisons relative to baseline values.

The upper two rows of Table 1 and Figure 1 present the measurements of the modified Griffith's scale and hemi‐MASI taken during the study. RM‐ANOVA showed significant group‐by‐time interactions for both the modified Griffith's scale (*p* = 0.01) and hemi‐MASI scores (*p* < 0.001). In the CEF + RGN6 group, the modified Griffith's scale scores dropped from 23.2 ± 5.14 at baseline to 16.4 ± 3.81 at Week 8, a 29.3% improvement (Figure 1A, *p* < 0.001). Likewise, hemi‐MASI scores in this group decreased from 2.93 ± 0.81 to 1.84 ± 0.73, reflecting a 37.2% improvement (Figure 1B, *p* < 0.001).

**TABLE 1:** Comparative analysis of skin aging parameters throughout the study period.

Parameters	Mean ± SD (% change)**				*p* ^†^
	Baseline	Week 2	Week 4	Week 8	RM‐ANOVA
Modified Griffith's scale					
CEF + RGN6	23.2 ± 5.14	22.3 ± 4.70 (−3.88%)	18.5 ± 3.77 (−20.3%)	16.4 ± 3.81 (−29.3%)	**0.01** *
CEF	23.2 ± 5.23	22.8 ± 5.01 (−1.72%)	20.1 ± 4.25 (−13.4%)	18.7 ± 4.13 (−19.4%)	
*p*‐value^‡^	> 0.99	0.68	0.15	**0.04** *	
Hemi‐MASI					
CEF + RGN6	2.93 ± 0.81	2.78 ± 0.74 (−5.12%)	2.15 ± 0.75 (−26.6%)	1.84 ± 0.73 (−37.2%)	**0.001** *
CEF	2.90 ± 0.82	2.87 ± 0.82 (−1.03%)	2.46 ± 0.73 (−15.2%)	2.31 ± 0.72 (−20.3%)	
*p*‐value^‡^	0.90	0.70	0.14	**0.02** *	
Skin elasticity (%)					
CEF + RGN6	49.2 ± 4.94	49.6 ± 5.00 (+0.81%)	52.4 ± 5.50 (+6.50%)	53.3 ± 5.03 (+8.33%)	0.65
CEF	48.5 ± 7.02	48.7 ± 6.66 (+0.41%)	50.9 ± 6.59 (+4.95%)	52.1 ± 7.00 (+7.42%)	
*p*‐value^‡^	0.67	0.58	0.36	0.48	
Skin hydration (AU)					
CEF + RGN6	72.0 ± 9.3	73.1 ± 9.6 (+1.53%)	79.6 ± 7.6 (+10.6%)	81.7 ± 7.8 (+13.5%)	0.72
CEF	72.7 ± 10.0	73.3 ± 8.8 (+0.83%)	78.7 ± 5.8 (+8.25%)	79.5 ± 9.9 (+9.35%)	
*p*‐value^‡^	0.80	0.93	0.64	0.39	
Melanin index (AU)					
CEF + RGN6	88.4 ± 28.5	85.3 ± 29.0 (−3.51%)	73.9 ± 24.6 (−16.4%)	70.5 ± 26.6 (−20.3%)	0.23
CEF	89.4 ± 25.9	85.2 ± 25.9 (−4.70%)	76.8 ± 24.5 (−14.1%)	75.7 ± 25.2 (−15.3%)	
*p*‐value^‡^	0.90	0.99	0.68	0.47	
Erythema index (AU)					
CEF + RGN6	180.0 ± 65.0	177.0 ± 53.4 (−1.67%)	166.0 ± 56.8 (−7.78%)	168.0 ± 61.7 (−6.67%)	0.60
CEF	179.0 ± 64.0	171.0 ± 59.1 (−4.47%)	172.0 ± 55.7 (−3.91%)	168.0 ± 58.2 (−6.15%)	
*p*‐value^‡^	0.98	0.71	0.70	0.98	

Abbreviations: AU, artificial unit; MASI, Melasma Area and Severity Index.

^†^ Repeated Measures ANOVA (RM‐ANOVA), overall *p*‐values of group × time.

^‡^ Post hoc analysis of each time point by Student's *t*‐test with Bonferroni's correction.

* Statistically significant *p* values are in bold.

** Defined as (follow‐up measurement—baseline measurement) / baseline measurement × 100%.

Post hoc analysis of between‐group comparisons demonstrated that the CEF + RGN6 group exhibited significantly greater improvements compared to the CEF group at Week 8 for both the modified Griffith's scale (*p* = 0.04) and hemi‐MASI (*p* = 0.02). Regarding within‐group comparisons of the modified Griffith's scale relative to baseline values, the CEF group showed significant changes only at Weeks 4 and 8, whereas the CEF + RGN6 group demonstrated significant improvements as early as Week 2, prior to the first laser treatment and reflecting 2 weeks of topical application alone (Figure 1A, *p* < 0.001). For hemi‐MASI scores, both groups demonstrated statistically significant reductions from baseline at all evaluated timepoints: Week 2 (*p* < 0.01) and Weeks 4 and 8 (p < 0.001) (Figure 1B).

**FIGURE 2:**
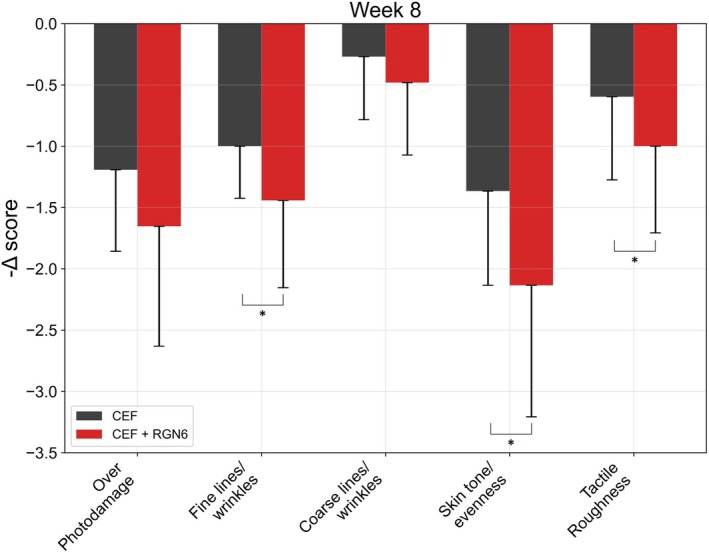
Subgroup analysis for the individual components of the Modified Griffith's scale at week 8. *p < 0.05 for comparisons between the CEF and CEF + RGN6 groups at week 8.

When individual components of the modified Griffith's scale were assessed at Week 8, the CEF + RGN6 group demonstrated significantly superior improvements in fine lines/wrinkles, skin tone evenness, and tactile roughness (Figure 2, *p* < 0.05). Throughout the study period, participant satisfaction scores showed statistically significant improvements from baseline at all timepoints in both the CEF + RGN6 and CEF groups on post hoc analysis (Figure 1C, *p* < 0.001); however, between‐group comparisons revealed no significant differences at any timepoint.

### 3.3 Objective Skin Biophysical Parameters

**FIGURE 3:**
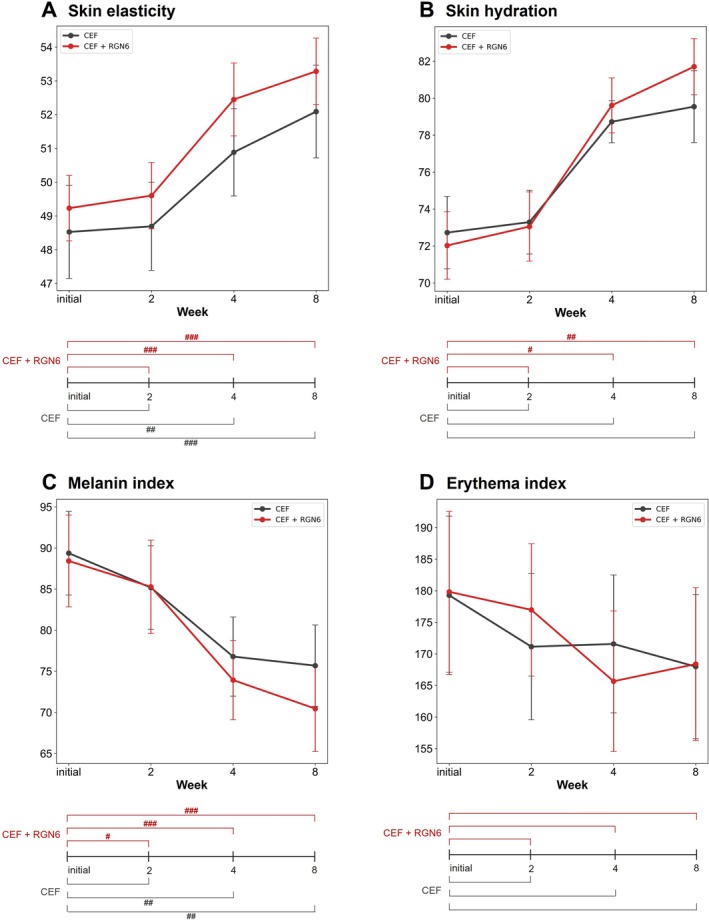
Temporal changes in skin biophysical parameters during 8 weeks of treatment: (A) Skin Elasticity, (B) Skin Hydration, (C) Melanin Index, and (D) Erythema Index. Values represent mean ± standard error. Upper graphs illustrate changes in clinical parameters over time, whereas lower bars indicate the statistical significance of differences between baseline and each time point. *p < 0.05, **p < 0.01, ***p < 0.001 for comparisons between CEF and CEF + RGN6 groups at each time point with Bonferroni correction, and #*p* < 0.05, ##*p* < 0.01, ###*p* < 0.001 for within‐group comparisons relative to baseline values.

The lower four rows of Table 1 and Figure 3 illustrate changes in skin biophysical parameters over the study period. RM‐ANOVA analysis did not reveal significant group‐by‐time interactions for any of the measured biophysical parameters. Additionally, between‐group comparisons (CEF versus CEF + RGN6) showed no statistically significant differences for any of the four measured parameters at any time point.

Regarding skin elasticity, the CEF + RGN6 group demonstrated an 8.33% improvement from baseline to Week 8, while the CEF group showed a 7.42% improvement (Figure 3A). Post hoc within‐group analysis indicated that both groups exhibited statistically significant enhancements in skin elasticity compared to baseline at Weeks 4 and 8 (*p* < 0.001).

For skin hydration, post hoc within‐group analysis revealed that the CEF + RGN6 group showed statistically significant improvements from baseline at Weeks 4 and 8, achieving 13.5% improvement at Week 8. In contrast, the CEF group, which showed 9.35% improvement at Week 8, did not demonstrate statistical significance at any time point (Figure 3B).

Regarding pigmentation parameters, post hoc within‐group analysis demonstrated that melanin index values in both the CEF + RGN6 and CEF groups showed statistically significant reductions from baseline at Weeks 4 and 8, with improvements of 20.3% and 15.3% at Week 8, respectively (Figure 3C, *p* < 0.001 and *p* < 0.01). Notably, the CEF + RGN6 group also exhibited a significant improvement in the melanin index from baseline to Week 2 (*p* < 0.05), whereas the CEF group did not show significant changes until Week 4.

For the erythema index, both groups demonstrated reductions of 6.67% and 6.15% at Week 8 for the CEF + RGN6 and CEF groups, respectively; however, these changes did not reach statistical significance in any analyses (Figure 3D).

### 3.4 Adverse Events

The treatment procedures and test products were generally well‐tolerated throughout the study period. Following Nd:YAG laser treatments, participants experienced transient erythema that resolved spontaneously within hours. After CO_2_ laser treatments, temporary crusting was observed, which resolved without intervention within several days. No contact dermatitis or adverse reactions attributable to the topical products were reported during the study. Additionally, no cases of post‐inflammatory hyperpigmentation were observed following laser treatments. All participants completed the study protocol without any treatment discontinuations due to adverse events.

## 4 Discussion

In this study, the combined post‐procedural application of an antioxidant serum and a recovery cream was associated with greater improvement in facial pigmentation and photoaging compared with antioxidant treatment alone over an 8‐week period. The combination regimen resulted in significant improvements in modified Griffith's scale scores and hemi‐MASI, with changes observed as early as Week 2 and becoming more pronounced by Week 8. Notably, pigmentation‐related outcomes consistently favored the combination‐treated side across multiple assessment modalities, including hemi‐MASI, skin tone evenness on the modified Griffith's scale, and objective melanin index measurements. Solar lentigines are associated with epidermal thickening and hyperkeratotic changes on histopathology [13]. When hyperkeratosis was clinically evident, focal CO_2_ ablation was performed in addition to pigment‐targeting treatment. The two‐week interval was supported by the mild recovery profile observed in this cohort.

The two‐week interval between sessions was considered appropriate given the limited downtime associated with picosecond treatment and the rapid resolution of CO_2_‐related crusting observed in this cohort. In our study population, erythema was mild and transient, and no delayed recovery or pigmentary worsening was noted, supporting the feasibility of this treatment schedule.

Post‐laser inflammation and oxidative stress are well‐recognized triggers of PIH, particularly in individuals with darker skin phototypes [14]. Antioxidant‐based post‐procedural care has therefore been proposed to attenuate these pathways and support early recovery. Previous clinical studies support this concept. For example, the addition of a topical antioxidant formulation containing silymarin, L‐ascorbic acid, and ferulic acid to non‐ablative laser treatment significantly reduced PIH and intralesional melanin compared with laser treatment alone [15]. Similarly, post‐laser use of a moisturizer enriched with anti‐inflammatory ingredients was shown to reduce erythema and downtime without increasing the risk of PIH [16, 17]. In line with these observations, the combination‐treated side in the present study demonstrated greater pigment clearance and more rapid clinical recovery at 8 weeks compared with antioxidant treatment alone, suggesting that enhanced post‐procedural antioxidant care may translate into improved pigmentation outcomes.

The superior outcomes observed with the combination regimen may be attributed to the complementary actions of additional active components in the recovery cream, which extend beyond the antioxidative effects of the serum alone [17]. These components target multiple aspects of post‐procedural recovery, including modulation of inflammation, restoration of the epidermal barrier, and support of dermal repair processes. Niacinamide has been reported to reduce melanosome transfer and downregulate inflammatory mediators, thereby contributing to the prevention of post‐inflammatory hyperpigmentation and improvement of uneven skin tone [18, 19, 20]. Ectoin and botanical‐derived anti‐inflammatory compounds have demonstrated protective effects against procedure‐induced erythema and oxidative stress, while also supporting hydration [16, 17, 21]. In addition, barrier‐supportive ingredients, including ceramide analogues and carbohydrate‐based repair agents, may facilitate re‐epithelialization and improve barrier integrity during the early post‐laser period. Signal peptides have also been investigated for their role in promoting dermal matrix remodeling, potentially contributing to improvements in skin texture and firmness following energy‐based procedures [11, 17, 22]. Consistent with these mechanisms, previous clinical reports have shown that post‐procedural formulations incorporating anti‐inflammatory and barrier‐repair ingredients are associated with faster resolution of erythema, improved skin tone evenness, and high patient satisfaction. Collectively, these findings support a multi‐mechanistic post‐laser care strategy that addresses inflammation, barrier recovery, and pigmentation pathways in parallel, rather than relying on antioxidant activity alone.

This study has several limitations that warrant consideration. First, the overall follow‐up period of 8 weeks indicates that the final outcome assessment occurred approximately 4 weeks after the second laser session, primarily reflecting short‐term changes in skin quality. As definitive pigmentary outcomes typically require longer observation (≥ 12 weeks), future studies with extended follow‐up are warranted to confirm the durability of the treatment effects. Second, as a single‐center study conducted exclusively in Korean participants with Fitzpatrick skin types III‐IV, these findings may have limited generalizability to other ethnic populations and different skin phototypes, particularly given the well‐established ethnic variations in pigmentary responses to laser treatments and topical agents. Third, despite the randomized split‐face design and standardized measurement protocols, potential confounding factors such as seasonal variations in UV exposure, individual differences in product application technique, and minor variations in measurement locations between visits may have influenced the skin biophysical parameters assessed in this study. Fourth, the study used a single‐blind design with blinded evaluators but not blinded participants, which could introduce bias into subjective assessments, such as the patient satisfaction questionnaire. However, the primary efficacy endpoints were assessed by blinded investigators using standardized photographic evaluation, and objective biophysical parameters were measured using validated noninvasive instruments, which are less susceptible to participant expectation bias. Fifth, the study population included heterogeneous pigmentary disorders with distinct pathogenetic mechanisms, which may limit the comparability of treatment outcomes across lesion types.

## 5 Conclusion

In conclusion, this split‐face randomized study demonstrates that an integrated post‐procedural skincare regimen combining antioxidant support with recovery‐focused care provides greater improvement in facial pigmentation and photoaging following laser treatment than antioxidant use alone. The combination approach was associated with earlier and more pronounced clinical improvements, without additional safety concerns. However, these findings pertain to short‐term outcomes, and the long‐term durability and efficacy of this treatment protocol have yet to be confirmed. Further studies with longer follow‐up periods are necessary to validate the sustained benefits of this approach. These findings support the role of multi‐mechanistic post‐laser care strategies in optimizing pigmentation outcomes, particularly in individuals at increased risk for post‐inflammatory hyperpigmentation.

## Author Contributions

Drs. Jihee Kim, Jemin Kim, and Patricia Brieva contributed to the study concept and design. Ms. Hyun Kim and Dr. Jihee Kim did the subject enrollment, measurement, and data collection. Ms. Jihee Boo and Dr. Shinwon Hwang did the data validation and statistical analysis. Drs. Jihee Kim and Jemin Kim contributed to data interpretation and provided expert insight into the report's writing. Writing of the original draft was conducted by Dr. Jamal Mohammed Alqahtani and Ms. Jihee Boo. All the authors approved the final version of the manuscript.

## Funding

This work was supported by Yonsei University Industry 2025‐31‐0382; (L'Oreal USA) and by the Yonsei University Faculty Research, 6–2024‐0056 and 6–2023‐0098.

## Ethics Statement

The authors confirm that the ethical policies of the journal, as outlined on the author guidelines page, have been adhered to and that appropriate approval from the ethics review committee has been obtained. This study was reviewed and approved by the institutional review board of Yongin Severance Hospital (9–2025‐0014).

## Consent

All patients have provided written consent for participating in this study. Additionally, informed consent was secured from the subjects depicted in the figures of this study.

## Conflicts of Interest

Dr. Brieva is employed by SkinCeuticals.

## Supporting information


**Table S1:** Summary of Indication‐Specific Laser Parameters Used in the Study.

## Data Availability

The data that support the findings of this study are available from the corresponding author upon reasonable request.

## References

[1] T. Passeron , R. Genedy , L. Salah , et al., “Laser Treatment of Hyperpigmented Lesions: Position Statement of the European Society of Laser in Dermatology,” Journal of the European Academy of Dermatology and Venereology 33, no. 6 (2019): 987–1005. 30873649 10.1111/jdv.15497

[2] A. Nautiyal and S. Wairkar , “Management of Hyperpigmentation: Current Treatments and Emerging Therapies,” Pigment Cell & Melanoma Research 34, no. 6 (2021): 1000–1014. 33998768 10.1111/pcmr.12986

[3] J. Ying , Y. Zhang , Y. Qiu , and W. Xiang , “The Role of Epidermal Growth Factor‐Containing Topical Products on Recovery and Post‐Inflammatory Hyperpigmentation Prevention After Laser Surgeries: A Systematic Review and Meta‐Analysis,” Journal of Cosmetic Dermatology 23, no. 2 (2024): 382–390. 37853844 10.1111/jocd.16007

[4] O. Agbai , I. Hamzavi , and J. Jagdeo , “Laser Treatments for Postinflammatory Hyperpigmentation: A Systematic Review,” JAMA Dermatology 153, no. 2 (2017): 199–206. 27973642 10.1001/jamadermatol.2016.4399

[5] S. Chaowattanapanit , N. Silpa‐Archa , I. Kohli , H. W. Lim , and I. Hamzavi , “Postinflammatory Hyperpigmentation: A Comprehensive Overview: Treatment Options and Prevention,” Journal of the American Academy of Dermatology 77, no. 4 (2017): 607–621. 28917452 10.1016/j.jaad.2017.01.036

[6] I. T. Y. Wong and V. Richer , “Prophylaxis of Post‐Inflammatory Hyperpigmentation From Energy‐Based Device Treatments: A Review,” Journal of Cutaneous Medicine and Surgery 25, no. 1 (2021): 77–86. 32929988 10.1177/1203475420957633

[7] L. Chen , Y. Dong , L. Liu , M. Zhu , and X. Qin , “Achieving the Maximum Efficacy by Combining Non‐Ablative Fractional Laser With Vitamin C, Vitamin E and Ferulic Acid Serum for Skin Quality Improvement in Asian Population,” Journal of Cosmetic Dermatology 23, no. 5 (2024): 1592–1596. 38279196 10.1111/jocd.16203

[8] J. M. Pullar , A. C. Carr , and M. C. M. Vissers , “The Roles of Vitamin C in Skin Health,” Nutrients 9, no. 8 (2017): 866. 28805671 10.3390/nu9080866 PMC5579659

[9] J. Kim , J. Kim , Y. I. Lee , A. Almurayshid , J. Y. Jung , and J. H. Lee , “Effect of a Topical Antioxidant Serum Containing Vitamin C, Vitamin E, and Ferulic Acid After Q‐Switched 1064‐Nm Nd:Yag Laser for Treatment of Environment‐Induced Skin Pigmentation,” Journal of Cosmetic Dermatology 19, no. 10 (2020): 2576–2582. 32052907 10.1111/jocd.13323

[10] Y. T. Chen , C. C. Chang , C. R. Hsu , J. H. Shen , C. J. Shih , and B. S. Lin , “Combined Vitamin C Sonophoresis and Neodymium‐Doped Yttrium Aluminum Garnet (Ndyag) Laser for Facial Hyperpigmentation: An Outcome Observation Study in Asian Patients,” Indian Journal of Dermatology, Venereology and Leprology 82, no. 5 (2016): 587. 10.4103/0378-6323.182806 27212284

[11] M. H. Gold , A. Wilson , E. Makino , and R. Mehta , “Improvements in Skin Quality Parameters in Postmenopausal Participants After Use of Topical Growth Factor Serum,” Journal of Cosmetic Dermatology 22, no. 1 (2023): 236–244. 36237142 10.1111/jocd.15472

[12] C. Liu , J. Boo , H. Kim , et al., “A Double‐Blinded, Split‐Face Clinical Trial Evaluating the Effects of a Vitamin C, E, and Ferulic Acid Serum Combined With Microneedling on Facial Photoaging,” Clinical, Cosmetic and Investigational Dermatology 19 (2026): 565035. 41710525 10.2147/CCID.S565035 PMC12912124

[13] C. Praetorius , R. A. Sturm , and E. Steingrimsson , “Sun‐Induced Freckling: Ephelides and Solar Lentigines,” Pigment Cell & Melanoma Research 27, no. 3 (2014): 339–350. 24517859 10.1111/pcmr.12232

[14] A. Bin Dakhil , A. Shadid , and S. Altalhab , “Post‐Inflammatory Hyperpigmentation After Carbon Dioxide Laser: Review of Prevention and Risk Factors,” Dermatology Reports 15, no. 4 (2023): 9703. 38205425 10.4081/dr.2023.9703 PMC10777097

[15] J. K. Hu , R. L. Quinonez , V. Antasiuk , and J. Waibel , “Treatment of Acne Vulgaris‐Associated Post‐Inflammatory Dyschromia With Combination of Non‐Ablative Laser Therapy and Topical Antioxidants,” Journal of Drugs in Dermatology 23, no. 9 (2024): 769–773. 39231081 10.36849/JDD.8309

[16] A. Marini , K. Reinelt , J. Krutmann , and A. Bilstein , “Ectoine‐Containing Cream in the Treatment of Mild to Moderate Atopic Dermatitis: A Randomised, Comparator‐Controlled, Intra‐Individual Double‐Blind, Multi‐Center Trial,” Skin Pharmacology and Physiology 27, no. 2 (2014): 57–65. 23949258 10.1159/000351381

[17] A. Alexis , R. A. Beach , P. Brieva , et al., “Real World Case Series: Integrated Skincare With Advanced Rgn‐6 Serum,” Journal of Cosmetic Dermatology 24, no. Suppl 2 (2025): e70303. 40709746 10.1111/jocd.70303 PMC12291419

[18] T. Hakozaki , L. Minwalla , J. Zhuang , et al., “The Effect of Niacinamide on Reducing Cutaneous Pigmentation and Suppression of Melanosome Transfer,” British Journal of Dermatology 147, no. 1 (2002): 20–31. 12100180 10.1046/j.1365-2133.2002.04834.x

[19] E. C. Davis and V. D. Callender , “Postinflammatory Hyperpigmentation: A Review of the Epidemiology, Clinical Features, and Treatment Options in Skin of Color,” Journal of Clinical and Aesthetic Dermatology 3, no. 7 (2010): 20–31. PMC2921758 20725554

[20] A. B. Kimball , J. R. Kaczvinsky , J. Li , et al., “Reduction in the Appearance of Facial Hyperpigmentation After Use of Moisturizers With a Combination of Topical Niacinamide and N‐Acetyl Glucosamine: Results of a Randomized, Double‐Blind, Vehicle‐Controlled Trial,” British Journal of Dermatology 162, no. 2 (2010): 435–441. 19845667 10.1111/j.1365-2133.2009.09477.x

[21] J. Buenger and H. Driller , “Ectoin: An Effective Natural Substance to Prevent Uva‐Induced Premature Photoaging,” Skin Pharmacology and Physiology 17, no. 5 (2004): 232–237. 15452409 10.1159/000080216

[22] F. Gorouhi and H. I. Maibach , “Role of Topical Peptides in Preventing or Treating Aged Skin,” International Journal of Cosmetic Science 31, no. 5 (2009): 327–345. 19570099 10.1111/j.1468-2494.2009.00490.x

