# Supplementary material for: Combination Topical Antioxidant Protocol Enhances Laser‐Induced Improvement in Facial Pigmentation and Photoaging: A Prospective, Split‐Face Trial
Source: J Cosmet Dermatol. 2026 Jun 19;25(6):e70994. doi: 10.1111/jocd.70994 (PMC13281373; doi:10.1111/jocd.70994)
Supplement: Supplementary file 1 — Table S1: Summary of Indication‐Specific Laser Parameters Used in the Study. [file JOCD-25-e70994-s001.docx]

| **Supplementary Table 1. Summary of Indication-Specific Laser Parameters Used in the Study** | | | | | | |  |  |  |
| --- | --- | --- | --- | --- | --- | --- | --- | --- | --- |
| **Indication** | **Laser Type** | **Wavelength** | **Spot Size** | **Energy (J)** | **Frequency (Hz)** | **Passes** | **Overlap** | **Immediate Endpoint** | **Additional Treatment** |
| Solar lentigines | Picosecond Nd:YAG, CO2 | 532 nm | 4 mm | 0.3–0.4 J | 7 Hz | 2 passes | 10–20% | Lesion darkening with white frosting and faint erythema | CO₂ laser (single pass) when clinically indicated |
| Freckles | Picosecond Nd:YAG | 532 nm | 4 mm | 0.3–0.4 J | 7 Hz | 2 passes | 10–20% | Lesion darkening | None |
| Melasma | Picosecond Nd:YAG | 1,064 nm | 6 mm | 1.0–1.2 J  (low-fluence) | 5 Hz | 2 passes | 10–20% | Faint erythema | None |
